# Comparison of gluteus medius strength between individuals with obesity and normal-weight individuals: a cross-sectional study

**DOI:** 10.1186/s12891-021-04470-8

**Published:** 2021-06-25

**Authors:** Rafael Ratti Fenato, Allan Cezar Faria Araujo, Ana Tereza Bittencourt Guimarães

**Affiliations:** 1 Faculty of Medicine, Federal University of Paraná, Paraná Toledo, Brazil; 2 Voglia Ortopedia, Maranhão Street, 790, Room 09, Centro, Paraná 85801-050 Cascavel, Brazil; 3 Obesity and Bariatric Surgery Center, University Hospital of the Western Paraná State University, Cascavel, Paraná Brazil; 4 Center of Biological and Health Sciences, Western Paraná State University, Paraná Cascavel, Brazil

**Keywords:** Obesity, Hip, Muscle weakness

## Abstract

**Background:**

The hip abductor muscles, primarily the gluteus medius, play an important role in stabilizing the pelvis during gait. Gluteus medius weakness is associated with biomechanical changes and musculoskeletal disorders. Individuals with obesity can have great difficulty maintaining abductor muscular function due to being overweight and possibly experiencing a decrease in muscle mass. However, it is still unclear whether the musculature of person with obesity can compensate for these changes. Therefore, the aim of this study was to compare gluteus medius strength between individuals with obesity and normal-weight individuals using a digital hand-held dynamometer.

**Methods:**

Twenty-five participants with obesity (BMI > 35 kg/m^2^) were matched for sex, age, and height with normal-weight individuals. Gluteus medius strength was measured by a single examiner using a belt-stabilized hand-held digital dynamometer placed on the knee of the individuals positioned in lateral decubitus. Three measurements were recorded with rest intervals, and only the highest value measured for each limb was used for analysis. The differences between pairs were calculated, and the normality of the data was assessed using the Shapiro-Wilk test (*p* < 0.05). The matrices of the variables were standardized and analysed using principal component analysis (PCA).

**Results:**

For the strength variables (Newtons) on both sides, no significant differences were detected between the groups (*p* > 0.05). However, significant differences were detected in these variables between the groups (*p* < 0.05) when the measurements were normalized to body weight (Newtons/kilograms). PCA indicated that both the absolute and normalized values of strength are lower in participants with obesity than in normal-weight.

**Conclusions:**

These findings suggest that people with obesity could have the same or less strength (PCA) to move more mass, which may imply a relative weakness that induces functional limitations.

**Supplementary Information:**

The online version contains supplementary material available at 10.1186/s12891-021-04470-8.

## Background

Hip abductor muscles play an important role in stabilizing the pelvis during gait, which allows the body to effectively maintain balance and lower limb mobility [1]. This group of muscles includes the gluteus medius, gluteus minimus, and tensor fasciae latae, but the gluteus medius is the main hip abductor muscle [2, 3].

The magnitude of force required by the hip abductors to stabilize the pelvis is approximately 2.5 times the individual’s body weight [4], as confirmed by *in vivo* studies [5]. Thus, the strength of the abductor muscles together must be higher than the individual’s body weight. When there is enough strength to support the individual’s body weight, his or her gait pattern is normal, and the joints work properly. If weight overload or muscle weakness occurs, an adaptation of the upper body will be triggered in an attempt to bring the centre of gravity closer to the centre of hip rotation.

Maintaining the strength of the gluteal musculature can be very challenging for subjects with obesity. When a three-dimensional gait analysis of subjects with obesity was performed, larger hip adduction, associated with marked ankle eversion, was observed during the terminal stance and pre-balance phases [6]. These findings are similar to those found in individuals with missing gluteal musculature, which leads to a pathological gait pattern, defined as Trendelenburg gait [7], as well as reduced abduction strength, an external rotation tendency, and internal rotation weakness of the lower limbs [8]. There is clearly an imbalance, which has a negative influence on gait parameters [9]. These changes lead to anteroposterior and mediolateral instability of the upper body and thus functional limitations and a predisposition to injuries [10–12].

Both overweight and individuals with obesity still suffer from the metabolic effects of adipose tissue on the muscular system. A decrease in anabolic hormones, such as growth hormones [13], and an increase in proinflammatory cytokines alter muscle metabolism. Both factors affect the amino acid balance, neuromuscular activation, and signalling pathways in the caspase cascade [13–16]. Finally, a decrease in muscle mass establishes a condition called sarcopenic obesity [13], in which the inflammatory cytokines produced by visceral fat are able to alter muscle metabolism and trigger a vicious cycle involving degeneration and a reduction in skeletal muscle quality [17–19].

It is very important to assess gluteus medius strength in individuals with obesity in clinical practice. Weakness in this muscle is associated with not only biomechanical changes but also musculoskeletal system disorders, such as hip arthrosis, lower back pain, knee arthrosis, and patellofemoral syndrome [20–26].

We conducted this study to compare the strength of the main gait-stabilizing muscle of individuals with obesity and normal-weight individuals. The aims of this study were to measure the strength of abductor muscles, especially the gluteus medius, using a digital hand-held dynamometer and to compare two groups of matched individuals: participants with obesity and participants with normal-weight.

## Methods

The present study is observational, quantitative, analytical, and cross-sectional. The UNIOESTE Human Research Ethics Committee (#1.180.202) approved it in July 2015. The patients provided written formal consent in accordance with the rules of the ethics committee.

Individuals with obesity who were beginning ambulatory follow-ups at the Obesity and Bariatric Surgery Service in the Western Paraná University Hospital (Cascavel, Paraná, Brazil) were included in the study. These individuals were of both sexes, were aged 20 to 60 years old, had grade II and III obesity, and had a body mass index (BMI) higher than 35 kg/m^2^ [27, 28]. The exclusion criteria were pregnancy, an orthopaedic disease of the lower limbs, locomotor system pain or sequelae, paresthesia or weakness in the lower limbs, orthostatic or walking pain, heart disease, or other diseases with restricted functional capacity.

For comparison, a group of normal-weight individuals (control group) was recruited, with a BMI below 24.9 kg/m^2^, a value considered normal. They were matched with the individuals with obesity by sex, age and height.

For the calculation of the sample size, a large effect size (0.8) was assumed due to the homogeneity of the group of patients from the Obesity and Bariatric Surgery Service of HUOP, and a type I error equivalent to 0.05 and a power of analysis with Student’s t-test of 0.80 were used. Based on these parameters, a total sample size of 54 was needed.

A single examiner evaluated the participants in an attempt to prevent analysis bias between observers from affecting the results. Weight (kilograms, kg) and height (meters, m) were measured.

All subjects eligible for the research were evaluated according to the exclusion criteria by assessing their clinical history and targeted physical examination findings. Individuals with pregnancy, an orthopaedic disease of the lower limbs, pain or sequelae in the locomotor system, self-reported paresthesia or weakness in the lower limbs, orthostatic or walking pain, heart disease or other diseases with restricted functional capacity were excluded.

The physical screening examination included an assessment of sensory disturbances, passive leg elevation and an assessment of hip joint pain after hip flexion and internal rotation and knee flexion and extension. Individuals who exhibited any signs suggestive of a pathology related to the impairment of the locomotor system were excluded.

**Fig. 1:**
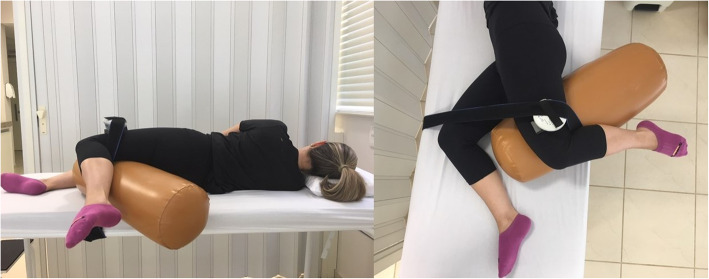
**a** Posterior and **b** superior view of the position used to measure gluteus medius strength

Gluteus medius strength was measured using a hand-held digital dynamometer (MICROFET2, Draper, USA), which has been shown to have high reliability in test-retest studies [29]. The device was positioned 5 cm proximal to the knee joint line, a technique adapted from the reports of Hislop and Montgomery [30–32]. The participant was positioned on a stretcher in lateral decubitus with a knee pad to avoid adduction, with slightly extended hips and anterior inclination of the pelvis, so that the strength components predominantly related to the gluteus medius could be measured (Fig. 1). The upper limbs remained relaxed to prevent them from affecting the strength test. The dynamometer was attached to the stretcher by using a rigid band, eliminating the need for the examiner to apply a resistance force. The participants were verbally asked to exert a maximum force against the device for 5 s, and after a 30-second rest interval, a new trial was performed. Three measurements were made, and only the highest value measured was used for analysis. After the analysis, the participant was repositioned for the measurement of the contralateral gluteus medius. The right side was always tested first.

### Statistical analysis

The intraclass correlation coefficient (ICC) was calculated to assess the reproducibility of the evaluator’s measurements. A pilot study was performed to evaluate the strength of both the right (RGM) and left (LGM) gluteus medius muscles of six selected individuals. Homogeneity of the measures was observed, with ICC values for the right and left sides equivalent to 0.9675 and 0.9288, respectively.

The data were tabulated in the Microsoft Excel 2013® program. Pairing was performed between groups by similarity in the variables sex, age, and height. The differences between pairs were calculated, and the normality of the data was assessed using the Shapiro-Wilk test. The comparisons between pairs were performed using the paired-samples t-test since all the variables were normally distributed. The statistical tests were performed using the R Core Team program (R Core Team, 2018) with a significance level of 0.05.

Then, matrices of the variables were standardized and analysed using principal component analysis (PCA). With PCA, the factor loads are defined as the correlations of each variable with the factor composition, where the factor is a new variable defined by the set of factor loads. This analysis did not consider the pairings but rather the subdivision of two large groups in an attempt to differentiate them. The factorial loads resulting from the main components were evaluated in terms of statistical significance using the independent-samples t-test.

## Results

**Fig. 2:**
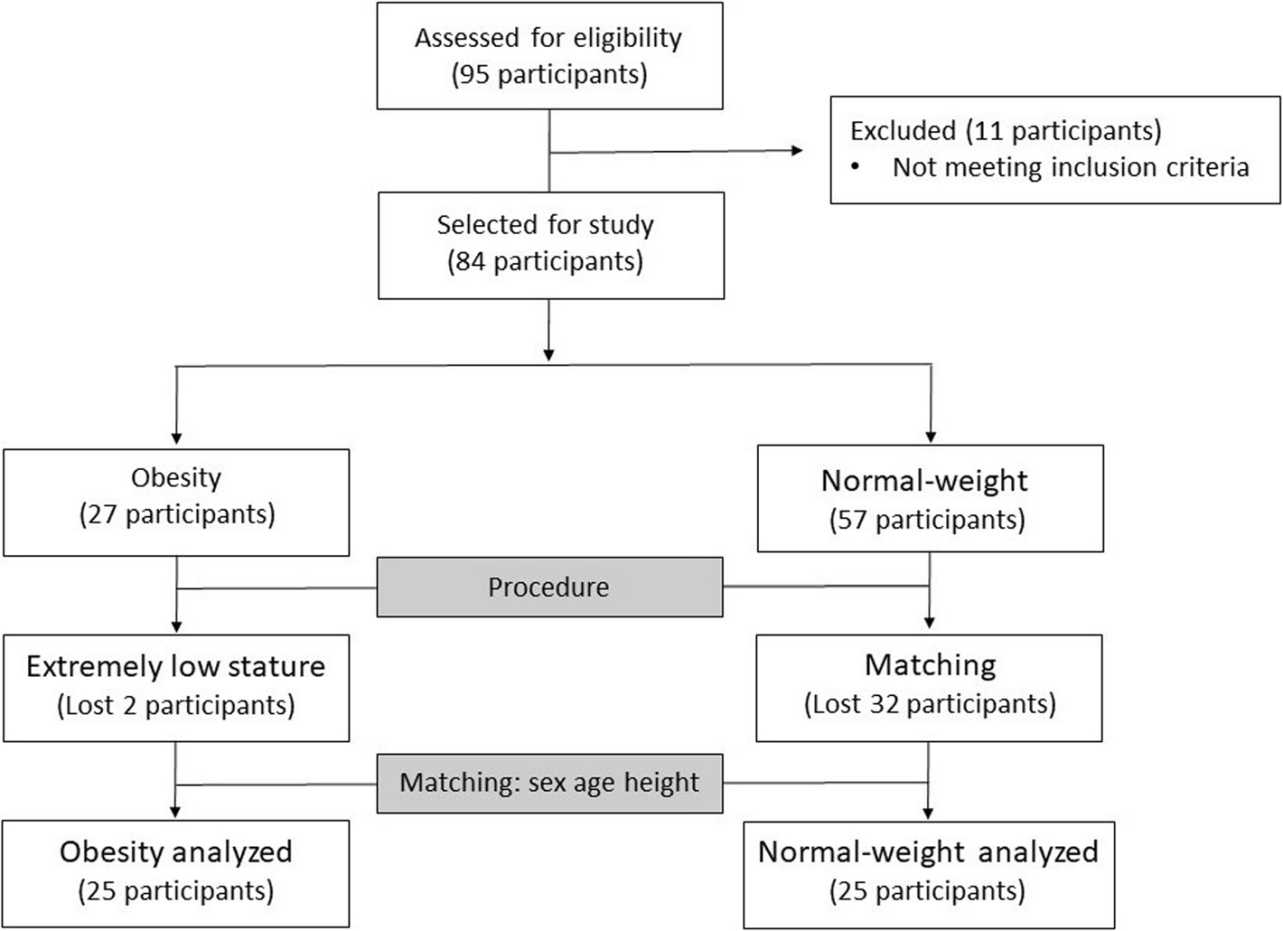
Diagrams showing a schematic summary of the participants recruited for this study

After the reliability of the measurements was assessed, 95 individuals were examined: 35 participants with obesity and 60 participants with normal-weight. Of these, 8 individuals from the obesity group and 3 individuals from the control group did not meet the selection criteria and were excluded. Two individuals with obesity were also excluded due to their short stature, which made it difficult to correlate their findings with the normal-weight individuals. Ultimately, 25 control-obesity pairs were formed, matched by gender and similarity in age and height. A total of 32 normal-weight participants were excluded because they could not be paired (Fig. 2).

Fifty individuals, including 4 (8 %) males and 46 (92 %) females, were included in the analysis, number that do not reach the recommended sample size because of the pairing. The variables of sex, age, and height were considered statistically equivalent between the obesity and control groups (*p* > 0.05). The weight and BMI variables showed statistically significant differences between pairs (*p* < 0.0001; Table 1). An additional file shows the data in more detail [see Additional file 1].

**Table 1:** Descriptive data of the pairs regarding age, height, weight, and BMI. *P*-value corresponding to the paired-samples t-test

Variable	Group	Minimum	Maximum	Mean	Standard deviation	***P***-value
Age	Obesity	20.000	60.000	43.600	9.785	0.407
	Control	23.000	57.000	42.880	10.647	
Height	Obesity	1.500	1.930	1.600	0.091	0.729
	Control	1.480	1.900	1.599	0.090	
Weight	Obesity	89.000	165.000	114.600	19.111	<0.0001*
	Control	45.000	80.000	58.068	8.825	
BMI	Obesity	36.616	56.008	44.604	5.126	<0.0001*
	Control	17.360	24.948	22.629	2.032	

**Fig. 3:**
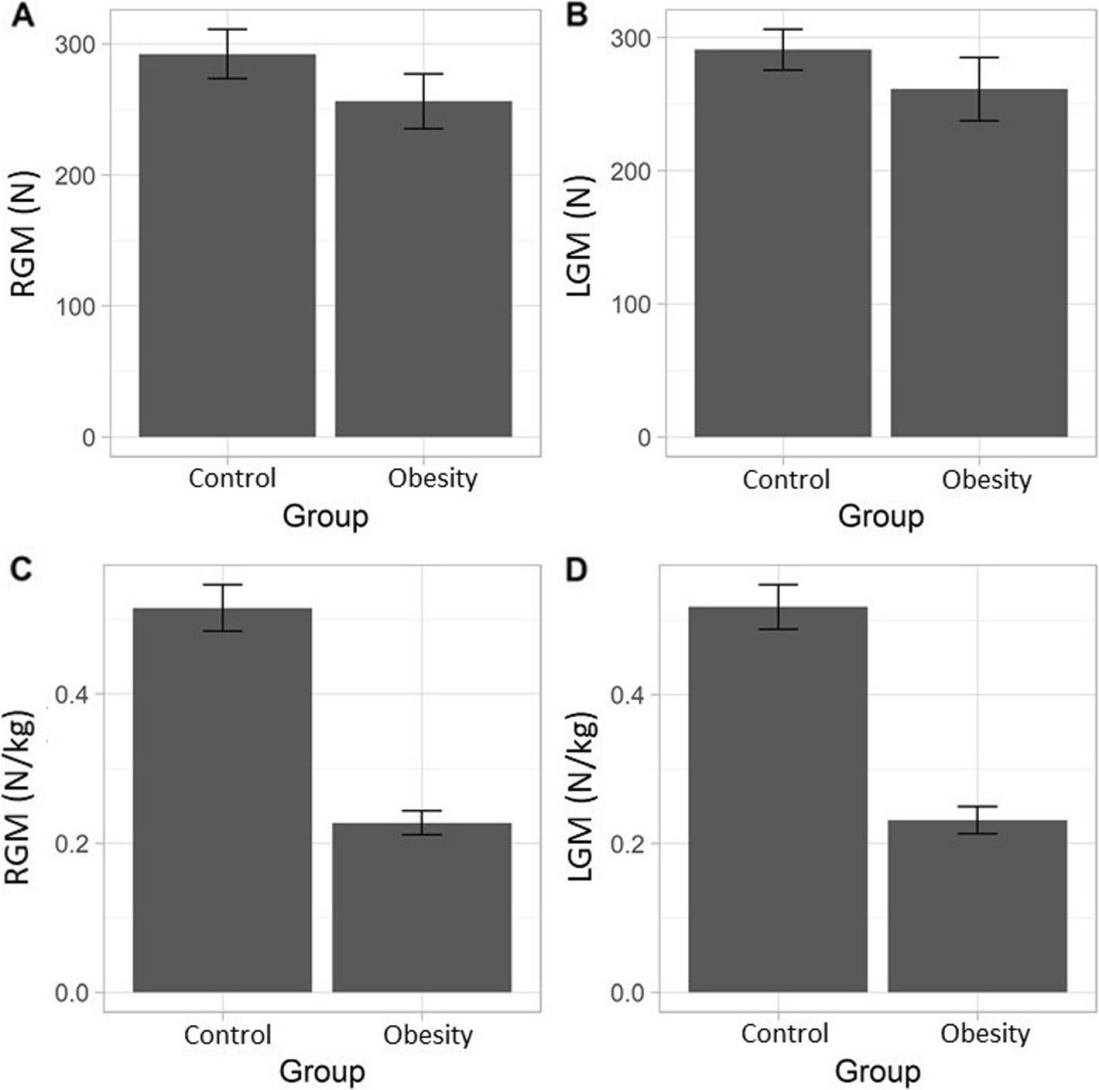
Comparative graphs showing the difference between the RGM and LGM. Legends: **a** RGM in N; **b** LGM in N; **c** RGM in N/kg; **d **LGM in N/kg

For the strength (N) of the RGM and LGM, no significant differences were detected between groups (*p* > 0.05). However, when the measurements were normalized to the body weight (N/kg), significant differences were detected in these to variables between groups (*p* < 0.05) (Table 2; Fig. 3).

**Table 2:** Descriptive results for the absolute strength and strength normalized to body weight of the RGM and LGM. *P*-value corresponding to the paired-samples t-test

		Control	Obesity	*P*-value
**Strength (N)**	RGM	292.0 ± 94.5	256.2 ± 104.2	0.149
	LGM	290.7 ± 76.6	261.1 ± 118.0	0.231
**Strength / Weight** **(N / Kg)**	RGM	51.5 ± 15.6	22.7 ± 8.0	< 0.0001
	LGM	51.8 ± 15.0	23.1 ± 9.1	< 0.0001

**Fig. 4:**
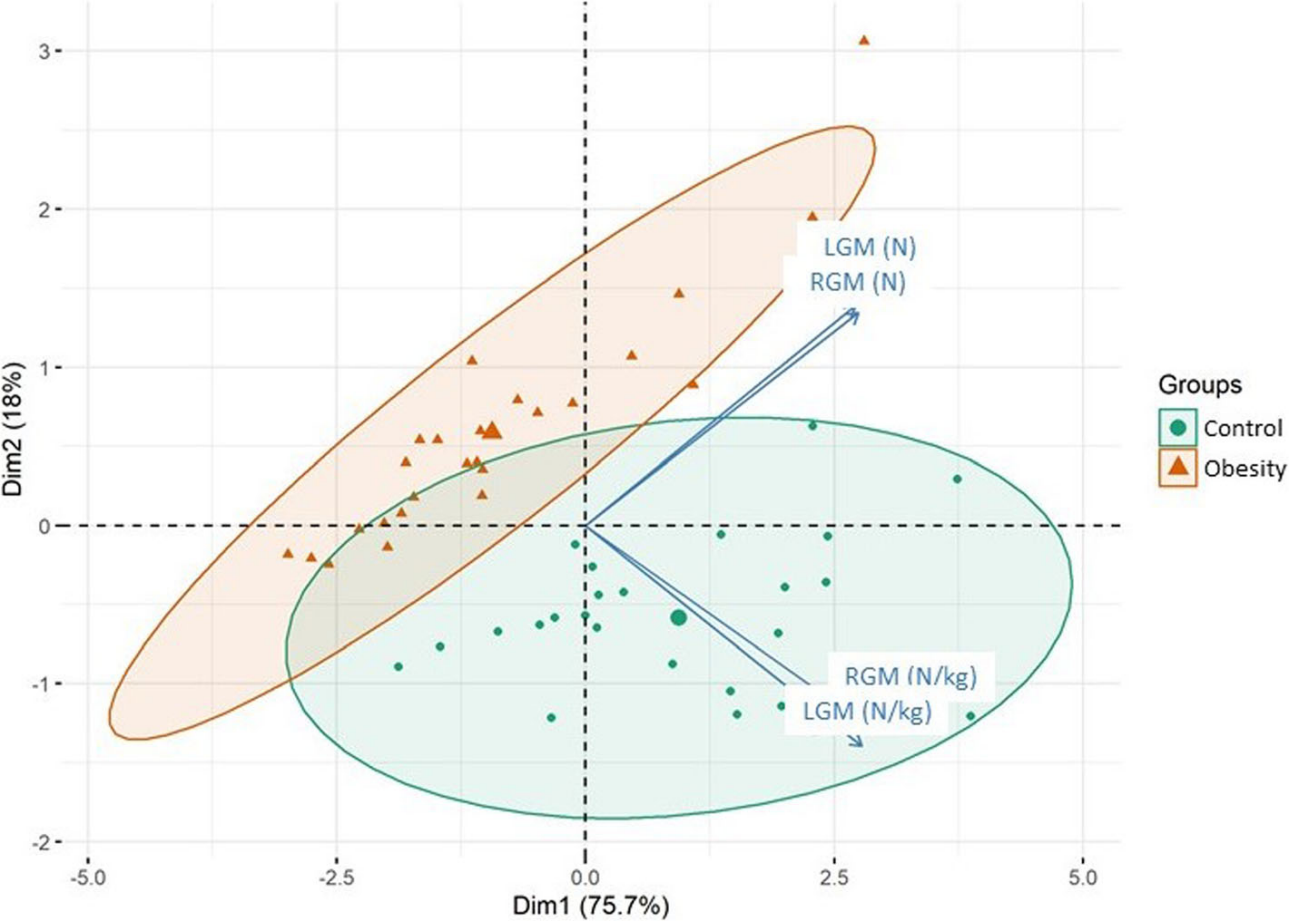
Ordering diagram of the principal components. Legends: RGM – right gluteus medius strength in N/kg; LGM – left gluteus medius strength in N/kg; RGM N – right gluteus medius strength in N; LGM N – left gluteus medius strength in N. Control (green ellipse) and obesity (orange ellipse) groups

Considering only the values normalized by weight among the individuals included in the analysis, the multivariate assessment verified the separation of the two groups: the control and obesity groups. In this study, pairing was not considered, only the division of two samples in relation to the force variable was considered to differentiate them. The first main component was defined as the variation in the strength of the RGM and LGM normalized to body weight (in N/kg) (eigenvalue = 3.03; variability = 75.67 %) and was directly related to the separation of the two groups analysed. The second main component was defined by the absolute muscle forces of the RGM and LGM (in N), which were also directly related (eigenvalue = 0.72; variability = 18.04 %; Fig. 4).

**Fig. 5:**
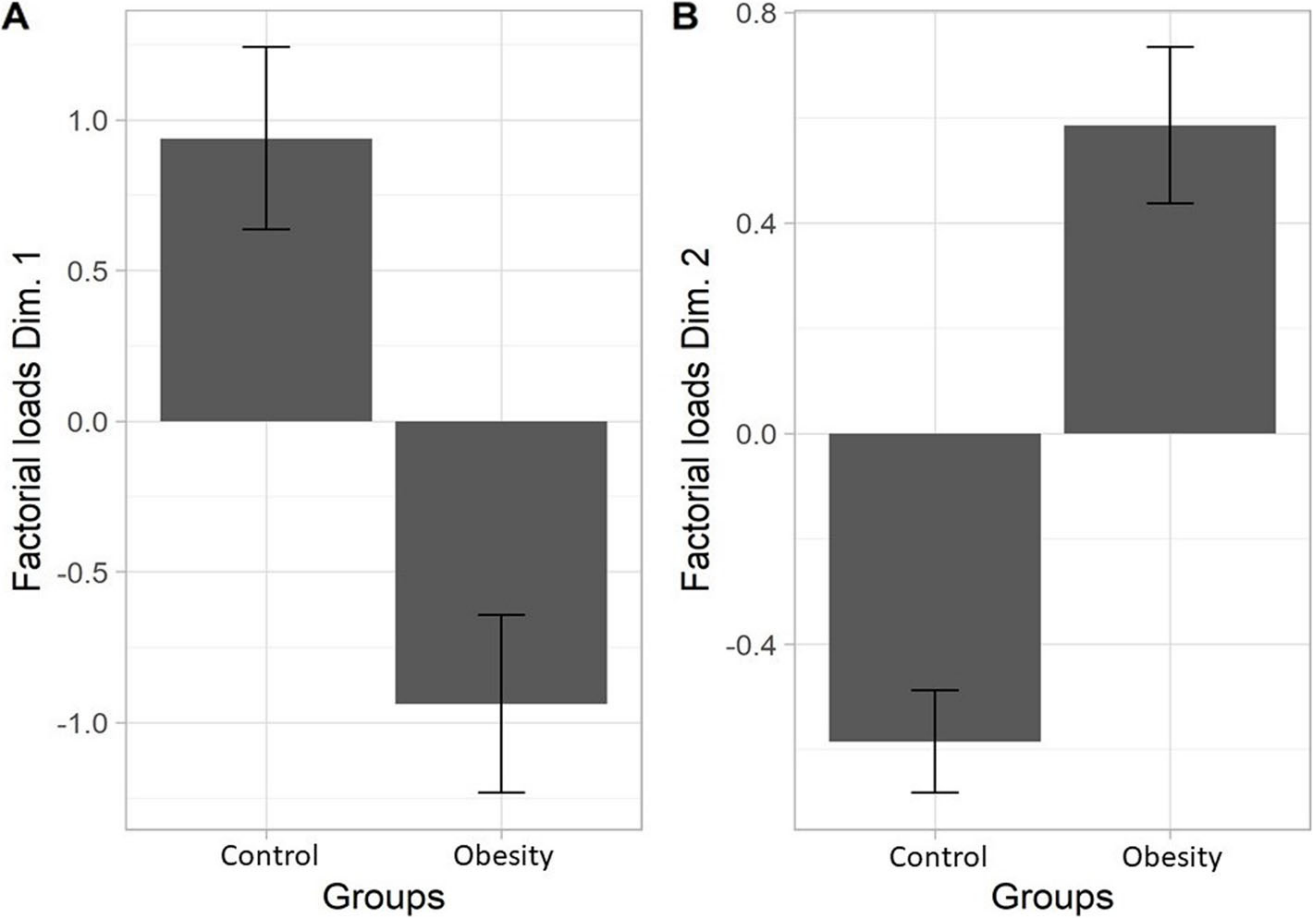
Means and standard errors of factor loads of the main components for the control and obesity groups. Legends: **a** first main component; **b** second main component

The factor loads of the main component 1, which represents the variation in muscle forces in N/kg, showed significant differences between pairs (*t* = 5.14; *p* < 0.0001; Fig. 5a), indicating that the strength values normalized to weight (in N/kg) of the RGM and LGM were higher in the normal-weight individuals. The factor loads of main component 2 also showed significant differences between groups (*t* = -8.63; *p* < 0.0001), indicating that the strength values of the RGM and LGM in N tend to be reduced in subjects with obesity (Fig. 5b).

## Discussion

Measuring the strength of hip muscles can be a difficult task. Several limitations have been reported regarding this task, including limited access to accurate equipment, difficulties in positioning the patient properly, variations in the support area on which the device is placed, the possibility of patient movement, and inconsistency in the verbal stimulus intensity [29]. Manual muscle testing is the most commonly used method for this purpose since it is easy and quick to perform, is free of charge, and does not require equipment [33]. Nevertheless, this test is subjective and descriptive, so it leads to low reliability and frequently overestimates the actual strength. The isokinetic dynamometer is the gold standard device for assessing muscle strength, with an exact and secure evaluation toll [34]. Because of the cost of an isokinetic testing device and difficulties in routine clinical testing, there is evidence that supports the clinical use of the hand-held dynamometer in routine medical examinations [35]. This device measures strength in an objective, precise and sensitive way [36]. However, the hand-held dynamometer is not without limitations, especially because this method is dependent on external adjustments to improve result validity and reliability [37]. It is a valid method when stabilized by a belt, and although these devices do not yield the same measures as isokinetic dynamometers, the values for hip muscle groups are correlated [38]. Thus, we chose to use a digital dynamometer stabilized by a brace to measure of the strength of the gluteus medius muscle.

The determination of the force required by the abductor muscles to balance the body in a standing position depends on two variables: pelvic anatomy and body weight [27]. In the present study, since individuals were paired in relation to sex, age, and height, it is assumed that there was a similarity in the pelvic anatomy between the pairs. Since the examination was performed by a single examiner, variability in the measurements due to differences in the technique used were not observed. Weight was the only relevant variable that could interfere with the strength data.

In the present study, individuals with obesity did not present a statistically significant difference in gluteus medius strength compared to normal-weigth individuals (*p* > 0.05). The absolute strength values were 292.0 N for the RGM and 290.7 N for the LGM in the control group. In the obesity group, the values were 256.2 and 261.1 N, respectively.

 In a literature review, Benfica et al. [28] reported the hip abductor muscle strength values in individuals aged between 50 and 59 years old to be 208.12 N for the dominant limb and 203.27 N for the nondominant limb in women and 305.97 N for the dominant limb and 298.49 N for non-dominant limbs in men. In the present study, this variation in the measurements can be explained by the age differences among the individuals included in the analysis, differences in sex, and differences related to the measurement technique.

The age range of the participants (from 20 to 60 years old) was chosen since it corresponds to an economically active group in whom movement disorders can greatly impact function and work. Additionally, individuals over 60 years of age may have reduced muscle mass and function [39].

It is worth noting that the study population in the present study consisted predominantly of women (92 %) for reasons of convenience and that abductor muscle strength varies between sexes. Women have lower abductor muscle strength, which corresponds to a higher risk of developing musculoskeletal pathologies [40].

In contrast to these findings in our study, some authors suggest that the antigravity muscles of subjects with obesity generate higher absolute forces [41–44]. Increased muscle strength is described as a beneficial adaptation to obesity, with excess body weight acting as a chronic training stimulus for daily activities [44].

Several studies have reported increased knee extension strength in individuals with obesity, with values varying from 10 to 30 % higher than those of normal-weight individuals [45]. However, gait analyses in individuals with obesity have shown a shorter stride length with a strategy involving quadriceps overloading and decreased hamstring activation [6, 46]. Due to gait changes, obesity can cause mechanical adaptations that favour the use of the strongest muscles and minimize the use of the weakest ones.

Regarding the gluteus medius, Lerner et al. [11] reported that subjects with obesity have higher absolute strength during gait and correlated this change with an increased BMI, reflecting the same theory of overload adaptation. These data were not confirmed by the gluteus medius strength analysis performed in the present study since there was no difference in the isometric strength values between the groups tested (*p* > 0.05). An analysis using nuclear magnetic resonance suggested that the gluteal musculature presents an increase in fat infiltrate as the BMI increases [47]. Although obesity increases muscle mass in the short term in young individuals, lipid infiltration in skeletal muscle can reduce the incorporation of amino acids into muscle proteins over time, with a decrease in total muscle mass [17]. It is possible that the long-term effect of obesity on muscle tissue overlaps with this weight stimulus on antigravity muscles and culminates in muscle loss over time [15]. This possibility may justify the findings of our study.

When the gluteus medius strength values were normalized to body weight, there was a significant difference (*p* < 0.05) between groups, which indicates that individuals with obesity have relative gluteus medius weakness compared to normal-weight individuals. Obesity results in larger and lower quality muscles, which have the same absolute strength and power as smaller muscles in thin individuals [48]. However, individuals with obesity generally struggle to move their body mass. A lack of strength can culminate in functional adaptations and imbalance, predisposing these individuals to injuries [10, 49].

Although some studies have suggested that individuals with obesity have higher absolute strength, they have less relative strength in some muscles, such as the quadriceps [50, 51]. Lafortuna et al. [52] also corroborated these data when they evaluated lower limb muscle strength through a leg-press exercise. Compared with normal-weight individuals, the subjects with obesity were stronger, but when the values were normalized by muscle mass, this difference disappeared.

When Lerner et al. [11] normalized the strength of the gluteal muscles by weight, there was no relevant difference between the obesity and normal-weight groups. Regarding muscle mass, the authors also reported that individuals with obesity required greater gluteal muscle strength for normal gait. This evidence is relevant since it suggests that individuals with obesity need stronger gluteal muscles, causing them to be more susceptible to fatigue. Thus, it was expected that overweight individuals have higher muscle strength to maintain balance while standing or walking. This fact was not proven by the results in the present study. When strength was normalized to body weight, the group with obesity had relative weakness in the gluteus medius muscle (*p* < 0.05). It can be concluded that strength alone does not seem to be an adequate parameter for assessing the abductor musculature since more than half of the world’s population is overweight and these strength values can be overestimated [53, 54].

The gluteal strength of individuals with obesity is a relevant factor since these two variables, obesity and weakness, are independently associated with musculoskeletal system changes [20–26, 55]. Moreover, according to new scientific evidence, muscle strength is inversely and independently associated with all-cause mortality [56]. Some authors even recommend the use of an algorithm to remove the dependence on body size and to more appropriately compare the strength of the hip muscles across individuals since it cannot be concluded that the force is directly proportional to body weight [57, 58].

When the statistical analysis of the factor loads was performed, it was possible to differentiate the two distinct groups for all gluteus medius force variables, regardless of whether they were normalized to body weight. This finding indicates that both the absolute strength values and those related to weight were different, constituting two distinct groups: the obesity group and the normal-weight or control group.

The present study has some limitations. First, despite the sample size being close to the recommended value in the sample calculation, we consider that it would be necessary to increase the number of subjects to reduce the effect size of the analysis. Therefore, additional studies are needed to confirm and increase the generalizability of the results found. Second, the study population was predominantly composed of women (92 %). Although this limitation did not interfere with the conclusions since the individuals were paired between groups, it would be interesting to increase the number of men since this sex are stronger than women. Activity level between groups should have been another variable and was not reported in this study. Additional studies are needed to prove whether there are morphological and functional changes in obesity gluteal muscles that may justify gait imbalances and associations with musculoskeletal disorders.

## Conclusions

The findings of the present study suggest that although subjects with obesity have the same absolute strength of the gluteus medius muscle as normal-weight individuals, when the strength is normalized as a function of body weight, it is possible to state that these individuals have such a weaker gluteus medius muscle. Since obesity is an epidemic, as the majority of the world’s population is overweight, it is recommended that individuals strengthen the gluteal muscles in relation to their weight, especially since obesity and weakness are independently associated with musculoskeletal system changes.

## Supplementary Information

**Additional file 1.** Data of paired pairs

## Data Availability

The datasets used and analysed during the current study are available in Additional file 1.

## Abbreviations

BMI: Body mass index
cm: Centimetres
ICC: Intraclass correlation coefficient
kg: Kilograms
LGM: Left gluteus medius muscle
m: Metres
N: Newtons
N/kg: Newtons per kilogram
PCA: Principal component analysis
RGM: Right gluteus medius muscle
UNIOESTE: Universidade Estadual do Oeste do Paraná
USA: United States of America
