# Supplementary material for: Comparison of gluteus medius strength between individuals with obesity and normal-weight individuals: a cross-sectional study
Source: BMC Musculoskelet Disord. 2021 Jun 25;22:584. doi: 10.1186/s12891-021-04470-8 (PMC8235575; doi:10.1186/s12891-021-04470-8)
Supplement: Supplementary file 1 — Additional file 1. Data of paired pairs [file 12891_2021_4470_MOESM1_ESM.docx]

| Pairs | Group | Age | BMI | RGM (N) | LGM (N) |
| --- | --- | --- | --- | --- | --- |
| 1 | Control | 40-50 | 24,78589055 | 56,4 | 39,6 |
| 2 | Control | 20-30 | 22,16 | 43,5 | 41,7 |
| 3 | Control | 50-60 | 24,03460984 | 38,2 | 38,2 |
| 4 | Control | 30-40 | 23,42209073 | 25,4 | 25,2 |
| 5 | Control | 40-50 | 20,93212012 | 32,4 | 27,3 |
| 6 | Control | 40-50 | 24,67702134 | 33,3 | 35,8 |
| 7 | Control | 30-40 | 24,55775234 | 25,2 | 25 |
| 8 | Control | 40-50 | 21,64412071 | 27,1 | 28,8 |
| 9 | Control | 30-40 | 24,8357635 | 26,3 | 29,3 |
| 10 | Control | 40-50 | 19,75232437 | 13,2 | 15,1 |
| 11 | Control | 30-40 | 24,6097337 | 26,8 | 27,3 |
| 12 | Control | 30-40 | 22,89281998 | 20,2 | 13,4 |
| 13 | Control | 20-30 | 23,78121284 | 25,2 | 28,7 |
| 14 | Control | 50-60 | 24,03460984 | 36,3 | 35,7 |
| 15 | Control | 50-60 | 22,49134948 | 42,4 | 37,1 |
| 16 | Control | 40-50 | 24,59937565 | 23,9 | 23,1 |
| 17 | Control | 50-60 | 21,34146341 | 23 | 17,4 |
| 18 | Control | 50-60 | 19,10525691 | 37,4 | 45 |
| 19 | Control | 50-60 | 20,88888889 | 28 | 31,1 |
| 20 | Control | 50-60 | 23,49523687 | 35,6 | 32,7 |
| 21 | Control | 50-60 | 24,9476697 | 19,5 | 25,7 |
| 22 | Control | 20-30 | 17,36044134 | 13,2 | 27,4 |
| 23 | Control | 20-30 | 23,05175491 | 26,8 | 26,1 |
| 24 | Control | 50-60 | 21,33 | 31,4 | 32,6 |
| 25 | Control | 40-50 | 21 | 33,8 | 31,8 |

Additional file 1

Data of paired pairs

| Pairs | Group | Age | BMI | RGM (N) | LGM (N) |
| --- | --- | --- | --- | --- | --- |
| 1 | Obesity | 40-50 | 43,7291334 | 42,9 | 55,8 |
| 2 | Obesity | 30-40 | 43,49110043 | 56,6 | 58,6 |
| 3 | Obesity | 50-60 | 44,00017087 | 12,2 | 14,4 |
| 4 | Obesity | 30-40 | 42,03648915 | 26,8 | 22,6 |
| 5 | Obesity | 40-50 | 48,01572045 | 29 | 29,6 |
| 6 | Obesity | 40-50 | 36,6163449 | 23,4 | 12,6 |
| 7 | Obesity | 40-50 | 50,54240631 | 36,9 | 43,5 |
| 8 | Obesity | 40-50 | 45,40988509 | 22,3 | 18,4 |
| 9 | Obesity | 30-40 | 50,98690716 | 19,5 | 22,7 |
| 10 | Obesity | 40-50 | 46,10636456 | 31,2 | 26,2 |
| 11 | Obesity | 30-40 | 40,08430878 | 26,9 | 22,4 |
| 12 | Obesity | 40-50 | 37,04474506 | 17,4 | 17,5 |
| 13 | Obesity | 30-40 | 54,49861276 | 25,6 | 29,8 |
| 14 | Obesity | 50-60 | 49,02202498 | 26 | 25,7 |
| 15 | Obesity | 50-60 | 42,49528233 | 15,2 | 9,3 |
| 16 | Obesity | 50-60 | 44,31607354 | 10,8 | 22,5 |
| 16 | Obesity | 40-50 | 39,19291624 | 27,4 | 20,8 |
| 18 | Obesity | 50-60 | 42,91760611 | 32,8 | 29,9 |
| 19 | Obesity | 50-60 | 39,46702648 | 10,2 | 17,8 |
| 20 | Obesity | 50-60 | 40,64766402 | 37,1 | 38,8 |
| 21 | Obesity | 50-60 | 41,2592803 | 19,8 | 18,7 |
| 22 | Obesity | 20-30 | 44,61848819 | 37,5 | 34,1 |
| 23 | Obesity | 20-30 | 51,12284355 | 24 | 21 |
| 24 | Obesity | 40-50 | 41,46666667 | 19 | 28,8 |
| 25 | Obesity | 40-50 | 56,00761773 | 22,6 | 24,2 |
